# Mechanical Measurement System and Precision Analysis for Tactile Property Evaluation of Porous Polymeric Materials

**DOI:** 10.3390/polym10040373

**Published:** 2018-03-27

**Authors:** Bao-Guo Yao, Yun-Liang Peng, Yun-Juan Yang

**Affiliations:** College of Mechatronics Engineering, China Jiliang University, Hangzhou 310018, China; 15757164756@163.com (Y.-L.P.); xbao_new2004@hotmail.com (Y.-J.Y.)

**Keywords:** measurement system, tactile properties, characterization, precision, evaluation, porous polymeric materials

## Abstract

Tactile properties are one of the most important attributes of porous polymeric materials such as textiles, comprising a subjective evaluation index for textile materials and functional clothing, primarily affecting the sensation of comfort during the wearing of a garment. A new test method was proposed, and a mechanical measurement system was developed to objectively characterize the tactile properties of porous polymeric materials by simulating the dynamic contact processes during human skin contact with the materials and in consideration of different aspects of tactile sensations. The measurement system can measure the bending, compression, friction, and thermal transfer properties in one apparatus, and is capable of associating the objective measurements with the subjective tactile sensations. The test and evaluation method, the components of the mechanical measurement system, the definition and grading method of the evaluation indices, and the neural network prediction model from objective test results to subjective sensations of tactile properties were presented. The experiments were conducted for the objective tests and correlation tests. Seven types of porous polymeric sheet materials from seven categories for the tactile properties were cut to a size of 200 mm × 200 mm and tested. Each index of tactile properties was significantly different (*P* < 0.05) between different sheet materials. The correlations of bending, compression, friction, and thermal transfer properties with Kawabata KES test methods were analyzed. An intra-laboratory test was conducted and an analysis of the variance was performed to determine the critical differences of within laboratory precisions of the measurement system. This mechanical measurement system provides a method and system for objective measurement and evaluation of tactile properties of porous polymeric sheet materials in industrial application.

## 1. Introduction

Comfort is the basic requirement in the daily life of human beings. Porous polymeric materials in sheet shape, such as textile materials, which are widely applied in clothing and household products, play an important role in wearing comfort and comfort sensations.

The handle properties of textile materials, which are defined as the attributes and performances of comfort sensations during contact between the human hand and the textile materials, have long been used in the clothing industries as the evaluation indices of product quality [1]. In the literature, extensive studies have been reported for handle property evaluation based on objective measurement of mechanical and physical performances and/or subjective evaluations [2,3,4,5,6,7,8,9,10,11,12]. The two most widely accepted and used test methods and measurement systems are Kawabata Evaluation System (KES) [2,3] and FAST (Fabric Assurance by Simple Testing) system [4,5]. The KES system, developed by Kawabata, consists of five instruments and is capable of simulating the handling process of human hand for the objective measurement of tension and shearing stress, bending, compression, surface friction, and thermal properties of textile samples. 

The FAST system is another set of instruments developed by CSIRO, Australia, and is basically a simplified version of the Japanese KES system. The FAST system is composed of four instruments and can measure the properties of compression, bending, tension and size stability of fabrics. These two test methods have to measure and characterize the handle properties of textile materials separately on several apparatuses and cannot take into consideration the interactions between different aspects of handle properties, and therefore do not have simultaneous perceptions of all aspects of handle. Based on the KES and FAST systems, fabric handle performances, including mechanical properties, have been investigated continuously in order to evaluate textiles and clothing comfort objectively [6]. The KES system was adopted in garment manufacturing to help to produce high quality clothing [7] and provided solutions and ways to develop fabrics based on the testing and analysis of handle properties [8,9]. A series of research works and methods developed based on the KES system have been reported, with some focusing on combining objective and subjective evaluations to obtain a regression model to predict fabric handle [10], some focusing on the measurement of certain mechanical properties such as bending rigidity and the relationship analysis between KES and FAST system [11], and some paying attention to automatic test process control [12]. Unlike the KES and FAST systems, which use several instruments, newly developed systems have been able to measure and evaluate the handle properties in one instrument [13,14,15], with the typical representative being the PhabrOmeter system developed by Pan [14]. The PhabrOmeter system applied the pattern recognition technique to evaluate handle properties and assess some visual attributes such as drape and wrinkle recovery, but the measured parameters and the test results did not have physical meanings.

Other studies have been conducted on the thermal and/or mechanical properties of polymeric materials, including porous polymers such as textile fabrics. These studies have mainly focused on the testing and characterization methods for thermal properties [16,17,18,19,20,21], tensile strength [22,23,24,25,26], friction properties [27,28,29], textural properties [30,31,32] and other mechanical properties of polymeric materials, such as bending strength and Izod impact strength [22], breaking tenacity [23] and compression performance [33]. For the measurement of thermal properties, typical methods include the calorimeter [16,17], thermistor [18], IR thermocamera [19], and heat flow meter bar approaches [20], as well as the transient plane source method by hot disk [21]. In the measurement of friction properties, the conventional method is tribometer [27,28]. There are three main types of tribometer probe and the contact arrangement: pin on disc, block on ring, and bouncing ball. Ramkumar et al. applied an artificial human finger sensor as a probe to measure friction in order to simulate the feeling mechanism of the fingers [29]. In the characterization of mechanical properties, Kismet took advantage of mechanical tests to address the influence of gamma radiation on mechanical properties such as tensile strength, bending strength and impact strength of powder coating reinforced polyolefin [22]. Ojstršek et al. developed a functional coating method to impact the mechanical, thermal and optical properties of the cotton fabric, which had the advantage of applying surface characterization to assess the changes in performance, including the tensile strength and breaking tenacity [23]. Manas et al. performed microindentation–tensile and tensile impact tests to survey and determine the changes of mechanical properties of thermoplastic polymers [24]. Lin et al. took advantage of an automatic textile stiffness tester to measure the softness of cationic fluorinated polymers [26]. These methods can be applied for the objective measurement and characterization of certain mechanical or thermal properties of porous polymeric materials, which are related to the handle properties.

Tactile property is a concept developed from handle property to denote the psychological sensations during the dynamic contact between the skin of the human body, including hand skin, and porous polymeric materials such as textiles. The tactile sensations are constituted from many aspects of subjective sensations, such as feelings of hardness, warmness, roughness and prickliness. Compared with handle performance, tactile properties for clothing comfort comprise a less-studied area, and existing test methods have mainly been focused on subjective evaluation methods [34,35,36,37] and the relational model between the tactility of certain subjective sensations and certain physical performances obtained from separate instruments [38,39]. Tactile properties have usually been subjectively evaluated by a panel of specialists [34], as well as by professional athletes [35], and volunteers through answering a questionnaire [36]. Based on the subjective evaluation results, Ramalho et al. compared the questionnaire answers for tactile perception with the results of the friction coefficient of friction measurements [36]. Park et al. improved the questionnaire method of subjective evaluation and developed a computerized textile sensibility evaluation system [37]. In the construction of the prediction model, a framework was proposed to address the correlations between tactile attributes, such as sensible texture and slipperiness, and the friction coefficient [38]. The objectively measured surface-fiber profile and geometrical roughness of fabrics were incorporated to predict the perceived softness and warmth of fabrics [39].

Although the existing test methods were capable of subjective evaluation of tactile properties, these subjective evaluation methods could not be applied to a large number of sample evaluations in industrial applications. The existing objective testing methods and the prediction models for tactile sense only provide certain physical parameters from certain instruments, and are not able to completely define dynamic interactions as complex as the contact process of tactile sensation and effectively correlate their objective measurements with tactile sensations. Therefore, the existing test methods are unable to measure and characterize the overall tactile properties in consideration of the main aspects of the tactile perception of porous polymeric materials such as textiles in a single instrument.

In order to objectively measure and evaluate the tactile properties of porous polymeric materials, a test method and an apparatus called Fabric Touch Tester (FTT) was proposed and developed [40]. However, when FTT was applied for industrial application and a large number of tests were carried out, it was found that the friction measurement method based on a friction disc was unreliable, inconvenient and difficult to maintain, and the instrument found it difficult to make consistent measurements. Moreover, FTT did not measure the displacement of the measuring heads, and could not obtain the actual compression work. Instead of the compression work, the product of the compression load and the time had to be applied, which had no physical meaning. Therefore, the test method and the mechanical device including the measurement components needed to be improved, especially with regard to the test methods for friction performance and compression properties. Meanwhile, the value of the evaluation index was not intuitive when classifying test samples in industrial applications, so the classification method also needed to be studied. The linear model of subjective and objective relations developed by Hu et al. in FTT was not able to effectively predict the commonality of subjective perception from the objective test data, and the prediction model had to be reconstructed to address the complex relationship between the objective measurements and the tactile sensations. It was necessary to modify the definitions of most of the evaluation indices, since the test methods had to be improved, and the original definitions of some indices did not have physical meanings. Furthermore, it was necessary to analyze the precision of the measurement system in order to determine critical differences of within-laboratory precision.

Therefore, a new test method was proposed and a mechanical measurement system called the Material Tactile Tester (MTT) was developed to objectively characterize the tactile properties of porous polymeric materials for the improvement of FTT in industrial applications. The test method, the evaluation indices definitions, the grading and classification methods, the subjective evaluation experiments and the prediction model were introduced in [41]. This paper focuses on the test and evaluation method, the design of the mechanical measurement system, the definition and grading of objective indices for tactile properties, the objective experiments, the correlations with Kawabata KES test methods, the intra-laboratory test, and the precision analysis of critical differences for single operator and within laboratory. The MTT test method and apparatus is able to measure and evaluate mechanical and physical properties simultaneously in one apparatus and quickly gives the evaluation results and the overall information on the tactile performance of porous polymeric materials.

## 2. Test and Evaluation Method

### 2.1. Mechanical Measurement System

**Figure 1:**
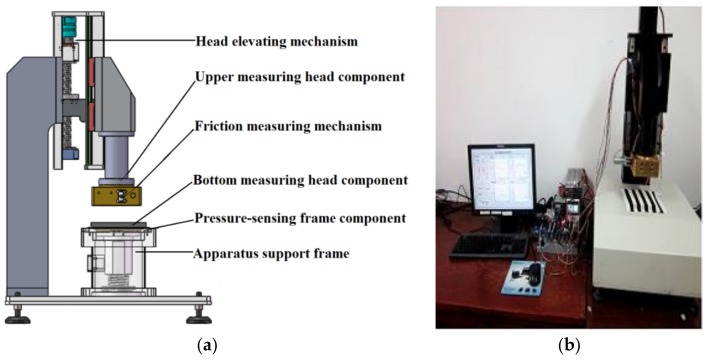
Mechanical device of the measurement system: (**a**) structural diagram; (**b**) prototype of the mechanical device.

As shown in Figure 1, the mechanical device of this measurement system comprises the following principal units: (1) Upper measuring head component; (2) Head elevating mechanism; (3) Pressure-sensing frame component; (4) Friction measuring mechanism; (5) Bottom measuring head component; (6) Apparatus support frame.

**Figure 2:**
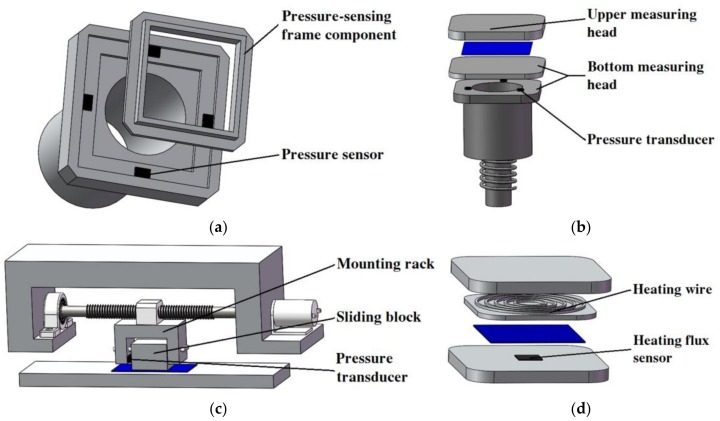
Integration measurement of tactile characteristics of four dimensions: (**a**) bending; (**b**) compression; (**c**) friction; (**d**) thermal transfer.

The mechanical measurement system is designed for the objective integrated measurement of tactile characteristics of four dimensions, including bending, compression, friction and thermal transfer properties, based on a mechanical device with a testing mechanism, a multi-sensory system, a virtual instrument data acquisition system and a motion control system (Figure 2).

The mechanical measurement system can simulate and implement the dynamic contact processes during contact between the skin of the human body and porous polymeric materials in sheet shape; and the porous polymer sheets used in textiles and household products are ideal test samples for this measurement system. Temperature, heat flux, pressure and displacement sensors are used in the multi-sensory system for the measurement of thermal transfer, surface friction, compression and bending signals during the dynamic contact process by the virtual instrument data acquisition system.

#### 2.1.1. Bending Measurement

The upper measuring head can move up and down in the vertical direction, and its motion is driven and controlled by the head elevating mechanism. A pressure-sensing frame component, which is installed on the bottom measuring head component, and the pressure transducers are applied to dynamically detect the bending force (Figure 2a). The sheet sample is laid on the bottom measuring head component. When the measurement begins, the upper measuring head moves downward, while the test sample edges are bent and exert some amount of pressure on the pressure-sensing frame component. Dynamic pressure changes are measured by the pressure sensors, and the signals are acquired by the computer as bending force changes vs. time through the data acquisition system to evaluate the bending properties of the test sample.

#### 2.1.2. Compression Measurement

**Figure 3:**
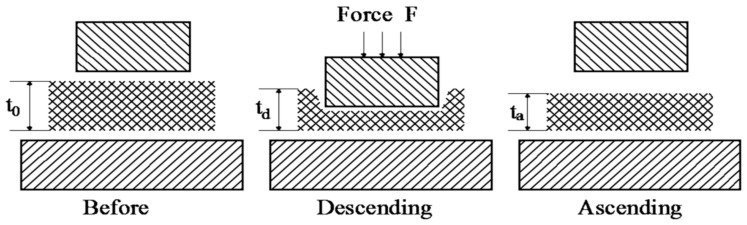
Measurement of compression properties.

The compression process of porous polymeric sheet materials includes two stages: the pressure stage of the upper measuring head descending, and the pressure release stage of the head ascending (Figure 3). There are three pressure transducers installed in the sandwich layer of the bottom measuring head, and the signals are averaged for the compression force measurement (Figure 2b). When the measurement begins, the upper measuring head is moved downward and the test sample is sandwiched between the two measuring heads. Because of the pressure applied, the pressure transducers detect the signals and the data acquisition system starts to acquire and record the test data as compression force changes vs. time. When the pressure reaches the set value, the motor of the head elevating mechanism stops, and the test sample is held in the compression sate between the two measuring heads. After two minutes, the motor begins to turn in reverse, and the upper measuring head ascends and returns to its original position automatically. Two displacement transducers are applied to measure the dynamic position changes of the two measuring heads to obtain the displacement of the compression force vs. time, as well as the sample thickness. Based on the compression force and displacement changes vs. time, the compression force vs. displacement can be obtained to evaluate the compression properties. Since the porous polymeric material sample is usually fluffy, the final thickness (*t*_a_) is always less than the initial thickness (*t*_0_).

#### 2.1.3. Friction Measurement

As shown in Figure 2c, the movements of the sliding block and the sliding block mounting rack are guided by two sliding block guide bars in the friction measuring mechanism. During the friction measurement, the bottom surface of the sliding block touches the test sample and the sliding block floats at the vertical direction. The movement of the sliding block is controlled by a DC motor, and the sliding block can move in a linear reciprocating mode on the test sample surface. The pressure transducer is installed on the side surface of the mounting rack between the sliding block and the mounting rack, which can be used to directly measure the friction force when the sliding block is in uniform moving. The friction force changes vs. time can be recorded by the data acquisition system to evaluate the friction properties of the test sample.

#### 2.1.4. Thermal Transfer Measurement

**Figure 4:**
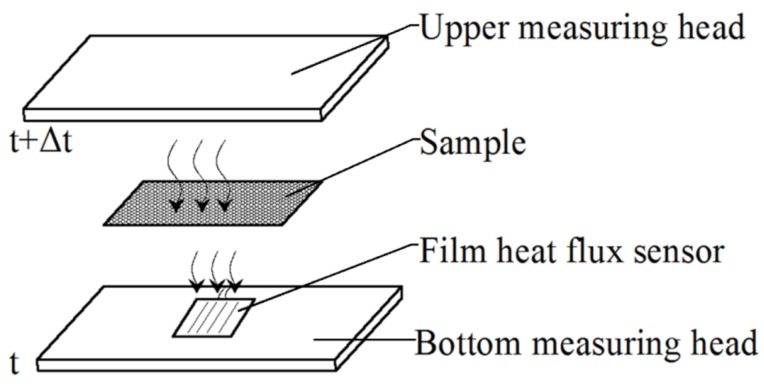
Schematic diagram of thermal transfer.

As shown in Figure 2d and Figure 4, there are six temperature sensors, which are distributed symmetrically between the two measuring heads for the measurement of the temperatures of the two surfaces of the test sample and the thermal transfer properties. A heating wire is set up inside the upper measuring head to warm the head in order to simulate the temperature difference between body skin and the sample during tactile sensations. A thin film heat flux sensor is installed on the surface of the bottom measuring head component and can be used to measure the heat passing through the test sample.

Before testing, the heating wire starts to work and the temperature sensors begin to acquire the temperature signals. When the temperature difference (∆*t*) between the upper measuring head and the bottom measuring head reaches 10 degrees centigrade, which represents the temperature difference between the environment and the outer surface of the human body in warm conditions, the heating wire stops heating. Then, the upper measuring head moves downward and touches the test sample placed on the surface of the bottom measuring head. Due to the temperature difference between the two measuring heads, the heat will flow through the test sample (Figure 4). The heat flow value can be detected by the heat flux sensor and acquired and saved as heat flux changes vs. time, together with the measured temperatures of the two surfaces of the sample, to evaluate the thermal transfer properties.

### 2.2. Objective Evaluation Indices and Grading Method of Tactile Properties

Derived from the source data and the signal curves, the evaluation indices were defined to objectively characterize the tactile characteristics of the porous polymeric materials.

#### 2.2.1. Thermal Transfer Properties

(1) Maximum heat flux value: $H_{M}$ (kW/m^2^)

(1) $$H_{M}={hf\left(t\right)|}_{max}$$

where *t* is the measurement time, and *hf*(*t*) is the heat flux change vs. time.

(2) Psychosensory intensity of descending stage: $PSI_{Des}$

$PSI_{Des}$ is the intensity of the thermal sensations during the descending process of the upper measuring head. $PSI_{Des}$ is defined as:

(2) $$PSI_{Des}=\int_{{th}_{1}}^{{th}_{2}}I\left(t\right)dt$$

where *I*(*t*) is the impulse due to the thermal stimulus and can be calculated from *hf*(*t*) and the temperature change rate of the upper measuring head based on Li’s method [42], *th*_1_ is the time when the heat begins to flow through the sample, and *th*_2_ is the time when the heat flow achieves steady status.

(3) Psychosensory intensity of ascending stage: $PSI_{Asc}$

$PSI_{Asc}$ is the intensity of the thermal sensations during the ascending process of the upper measuring head. $PSI_{Asc}$ is defined as:

(3) $$PSI_{Asc}=\int_{{th}_{3}}^{{th}_{4}}I\left(t\right)dt,$$

where *th*_3_ is the time when the heat flow begins to decrease from steady status, and *th*_4_ is the time at which the heat flow has just decreased to zero.

#### 2.2.2. Bending Properties

The indices of maximum bending force (BS_max_), bending rigidity (WBS_down_), and bending recovery rigidity (WBS_up_) defined in [40] were kept, and the index symbols were revised to $F_{BM}$, $W_{BD}$ and $W_{BA}$ accordingly.

#### 2.2.3. Friction Properties

(1) Static friction index: SFI

Static friction index (SFI) is calculated as the maximum friction force divided by the weight of the sliding block for fiction measurement.

(4) $$SFI=\frac{{Ff\left(t\right)|}_{max}}{W_{sb}}$$

where *Ff*(*t*) is the friction force signal against time, and *W_sb_* is the weight of the sliding block as the normal pressure since the sliding block floats at the vertical direction.

(2) Dynamic friction index: DFI

Dynamic friction index (DFI) is defined as the average friction force divided by the weight of the sliding block.

(5) $$DFI=\frac{\int_{0}^{T}Ff\left(t\right)dt}{W_{sb}\cdot T}$$

where *T* is the total measurement time of friction.

(3) Intensity index of friction: FII (N·s)

Intensity index of friction (FII) is defined as the integral of the friction force curve during the friction measurement.

(6) $$FII=\int_{0}^{T}Ff\left(t\right)dt$$

#### 2.2.4. Compression Properties

(1) Compression work of the pressure stage: $W_{CD}$ (mN·cm/cm^2^)

The compression work of the pressure stage ($W_{CD}$) is defined as the work done by the compression force on the unit area during the pressure stage.

(7) $$W_{CD}=\int_{D_{1}}^{D_{\max}}CF\left(x\right)dx$$

where *x* is the displacement of the compression force, *CF*(*x*) is the compression force on unit area vs. displacement, *D*_1_ is the displacement when *CF*(*x*) begins to rise from zero, and *D*_max_ is the displacement when *CF*(*x*) achieves its peak value.

(2) Compression work of the pressure release stage: $W_{CA}$ (mN·cm/cm^2^)

Compression work of the pressure release stage ($W_{CA}$) is defined as the work done by the compression force on the unit area during the pressure release stage.

(8) $$W_{CA}=\int_{D_{2}}^{D_{\max}}CF\left(x\right)dx$$

where *D*_2_ is the displacement when the compression force has just decreased to zero during the pressure release stage.

(3) Compression resilience index: CRI (%)

Compression resilience index (CRI ) is defined as the compression work of the pressure release stage ($W_{CA}$) divided by the compression work of pressure stage ($W_{CD}$).

(9) $$CRI=\frac{W_{CA}}{W_{CD}}\times 100\%$$

The defined indices are listed in Table 1.

In the development of testing standards and the promotion of the test method for industrial applications and quality control, it was necessary to grade the values of the evaluation indices of the test results. The indices for the objective evaluation of the tactile properties were converted from values to grades, using a five-grade (1, 2, 3, 4 and 5) method. The values of the indices in each of the five grades were determined on the basis of the grading test results of about one hundred samples of porous polymer sheets used in textiles and household products, which were purchased from department stores and included porous polymeric sheet materials with different structural parameters and made from different materials.

### 2.3. Prediction Model from Objective Test Results to Subjective Sensations

Twelve samples randomly selected from one hundred different samples of the grading test were conducted the objective and subjective tests using the same laboratory settings. Five specimens for each sample were tested. One hundred trained volunteers participated in the subjective tests. The number of male and female volunteers was equal. The volunteers were separated into four groups based on both the area of their hometown and their age. After evaluating each specimen, the volunteers recorded the scores (from one to five) of the contact perception of the four subjective indices such as hardness, warmness, roughness and prickle in the questionnaire form. The objective test and one-way ANOVA analysis results showed that each objective evaluation index was significantly different in tactile properties between different samples and the defined indices could differentiate the tactile properties of the test samples. Moreover, the subjective evaluation results showed that in general, the subjective sensations of hardness, warmness, roughness, and prickliness exhibited commonality within age, gender, and area factors.

On the basis of the objective and subjective test results, a back-propagation network (BPN) prediction model from objective test results to subjective sensations was built and tested. The twelve samples were separated into training group and test group in order to train and test the model. The objective evaluation indices were set as the neurons of input layer, and the four subjective indices of hardness, warmness, roughness and prickle were the output layer neurons. After training and testing of the prediction model, the results showed that the predictions of the BPN model agreed well with the subjectively evaluated values for the four subjective indices, and the relative errors were less than 6%. The BPN prediction model could be applied to predict the subjective sensations from the objective measurement. Therefore, the tactile properties of porous polymeric sheet materials can be objectively characterized by simultaneously measuring the bending, friction, compression and thermal transfer properties in one instrument, i.e., the MTT, in consideration of the interactions between different aspects of tactile sensations, and MTT is capable of associating the objective measurements with the subjective tactile sensations.

Based on the subjective index results and the classification method, the test samples were classified for their tactile properties into seven categories, in warm conditions, as warm and stiff, cool and stiff, soft-warm and rough, soft-warm and smooth, soft-cool and scratchy, soft-cool and rough, and soft-cool and smooth.

The prediction model and the classification method were reported in detail in [41].

## 3. Materials and Experiments

### 3.1. Experimental Protocol for Objective Test and Correlation Test with KES Test Method

The objective tests on tactile properties using MTT and the correlation tests using Kawabata KES system were carried out to evaluate seven samples (A–G) from seven categories of tactile properties, since the KES system is widely used and accepted in the field of testing and characterizing of mechanical and physical properties of porous polymeric materials.

Seven typical samples from seven categories, with one sample for each category, were selected from 100 different porous polymeric sheet materials for the grading tests and ordered by random numbers; they were cut to a size of 200 mm × 200 mm and measured in the objective test and correlation test. All the tests were carried out in a condition room, where the environmental conditions were controlled at 21 ± 1 °C and 65% ± 2% relative humidity according to ASTM D 1776 in order to avoid the influence of external factors such as environmental temperature and humidity on the objectivity of the experimental data. The content and structural parameters of the seven test samples are shown in Table 2.

The MTT system and the KES system with four instruments were used to conduct the objective and correlation tests of the seven samples, respectively. For each set of samples, five specimens were tested.

The objective indices of tactile properties for the correlation analysis between the MTT system and the KES system are shown in Table 3.

### 3.2. Experimental Protocol for Precision Study

The intra-laboratory tests for the precision study of the tactile properties of porous polymeric materials were conducted in one laboratory to evaluate four samples by two operators. The experiments were performed as follows:

(1) Four samples from four different materials;
(2) Ten specimens per sample (five specimens for each operator);
(3) Two operators.

The basic structural features of the four test samples are shown in Table 4.

All the tests of the intra-laboratory were carried out using the same experimental settings, including the environmental conditions used for the objective test.

## 4. Objective Test Results and Discussion

### 4.1. Objective Test Results

The test results of MTT and KES system by mean values are summarized in Table 5 and Table 6, respectively. Each index of tactile properties is significantly different (*P* < 0.05) between the different porous polymeric sheet materials.

**Figure 5:**
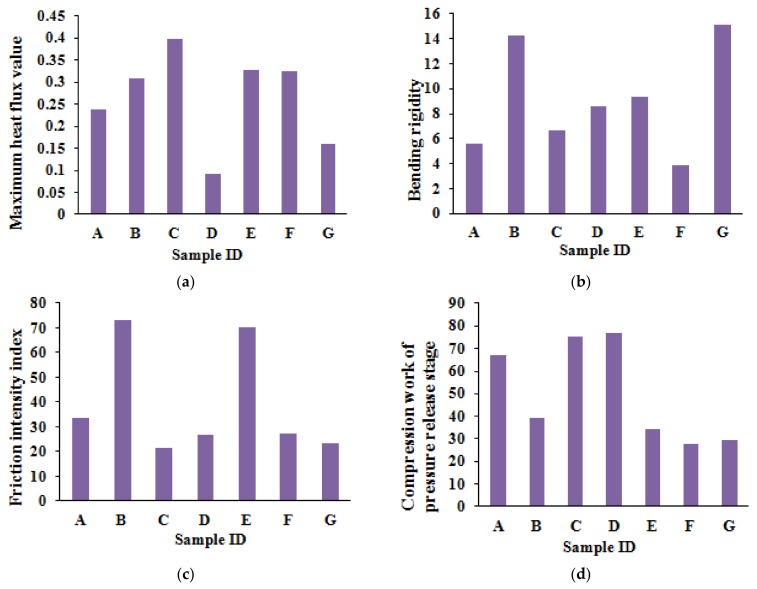
Histogram of MTT test results: (**a**) maximum heat flux value; (**b**) bending rigidity; (**c**) intensity index of friction; (**d**) compression work of pressure release stage.

The histogram graphs of MTT test results for the four indices—maximum heat flux value, bending rigidity, intensity index of friction, and compression work of pressure release stage—are shown in Figure 5. The heat conducting property of sample C is the best, since it has the largest value of maximum heat flux. On the other hand, sample D has the worst heat conducting property, with the smallest value of maximum heat flux. Sample G is the stiffest sample since it has the largest value of bending rigidity. Sample B has the largest value of friction intensity index, and sample C has the smallest value of friction intensity index, indicating that sample B is the roughest, while sample C is the smoothest. Moreover, sample D has the largest value of compression work. Therefore, sample D has the highest compression susceptibility.

Sample C is linen, and the surface contact sensation is cool and smooth. Therefore, Sample C has better heat conducting property, and the value of friction intensity index is smaller. Sample B is expanded polyethylene foam. The pores are larger, and the surface is relatively rough, so the friction intensity index of sample B is larger. Sample G certainly has a larger value for bending rigidity, since it is relatively stiff silicone rubber.

### 4.2. Correlations with KES Test Method

Using the test results of seven typical samples with tactile properties, the thermal transfer, bending, friction and compression properties of MTT testing were compared with the test results of the Kawabata KES test method. The correlations are significant (*P* = 0.00 < 0.05) when comparing maximum heat flux value *(*$H_{M}$), bending rigidity ($W_{BD}$), intensity index of friction (FII), and compression work of pressure release stage ($W_{CA}$) of the MTT tests in one instrument with the Kawabata KES tests for thermal conductivity (K), Bending moment (B), mean friction coefficient (MIU), and linearity of compression (LC) using four instruments.

**Figure 6:**
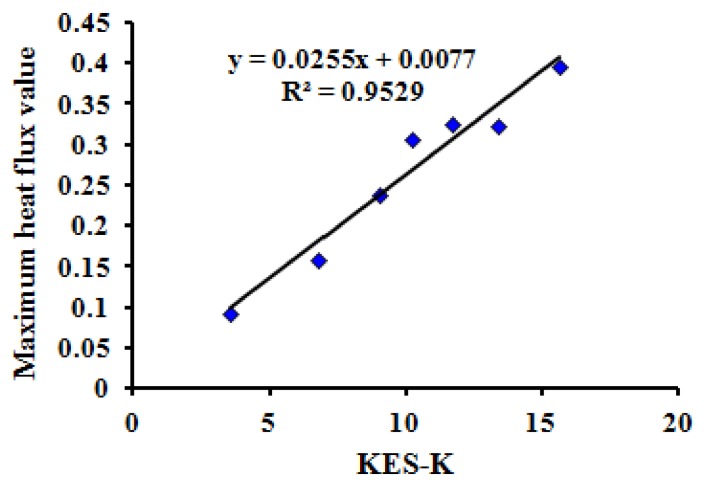
Relationship between maximum heat flux value ($H_{M}$) of MTT test and KES thermal test.

**Figure 7:**
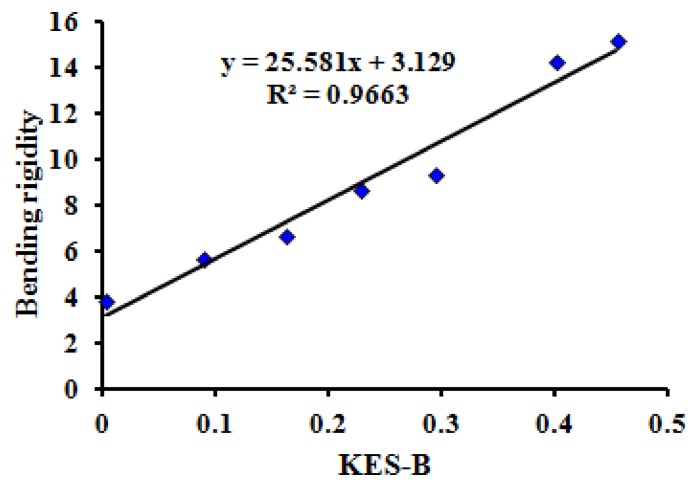
Relationship between bending rigidity ($W_{BD}$) of MTT test and KES bending test.

**Figure 8:**
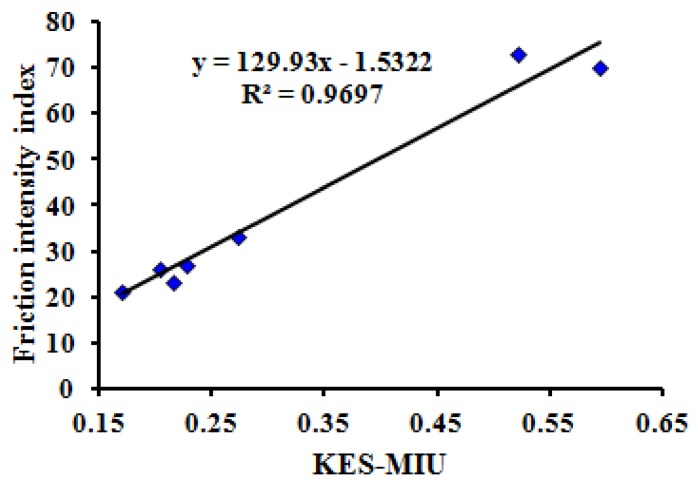
Relationship between intensity index of friction (FII) of MTT test and KES friction coefficient test.

**Figure 9:**
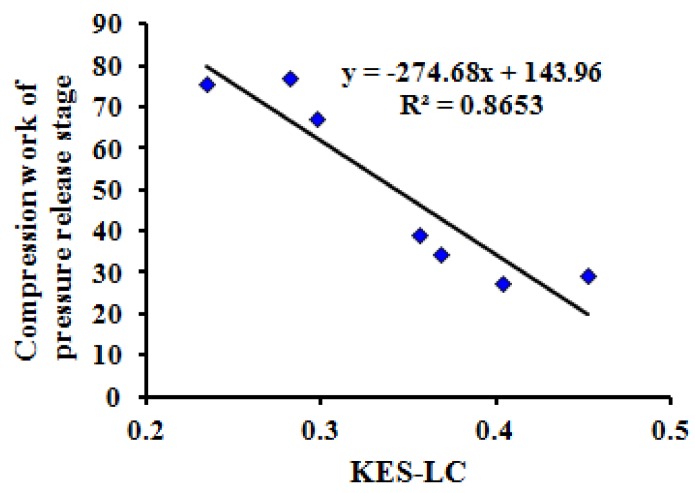
Relationship between compression work of pressure release stage ($W_{CA}$) of MTT test and KES compression test.

Figure 6, Figure 7, Figure 8 and Figure 9 show the relationship between the MTT tests and Kawabata KES tests.

The correlation coefficients ($R^{2}$) between the three indices ($H_{M}$, $W_{BD}$, FII) of the MTT test and the three indices (K, B, MIU) of the KES test are all more than 0.95. Meanwhile, the correlation coefficient ($R^{2}$) between $W_{CA}$ and LC is 0.8653. This reveals that the compared indices have linear correlations, and that the MTT test system can effectively achieve objective testing of tactile properties.

The KES system requires four instruments to complete the experiments, and therefore cannot provide simultaneous sensing of all aspects of handle properties. However, the MTT system uses only one instrument to carry out testing of the tactile properties of porous polymeric materials. MTT completely considers dynamic interactions as complex as the contact process of tactile sensation, and is capable of associating objective measurements with subjective tactile sensations of porous polymeric materials by using a grading method of the evaluation indices and a neural network prediction model.

## 5. Precision Analysis

An intra-laboratory test was carried out, and an analysis of variance for the mechanical measurement system MTT was conducted in accordance with the ASTM Standard D 2904 and ASTM Standard D 2906.

A critical difference is the observed difference between two test results, and can be calculated according to ASTM D 2906 as:

(10) $$\mathrm{Critical}\;\mathrm{difference}\;\mathrm{between}\;\mathrm{averages}\;\left(\mathrm{unit}\;\mathrm{of}\;\mathrm{measure}\right)=1.414zs_{T}$$

where z is the standard normal deviation for the prescriptive probability level (*z* = 1.960 for the 95% probability level); $s_{T}$ is the standard error and can be calculated using Equations (11)–(13):

(11) $$s_{T}\left(\sin\;\mathrm{gle}-\mathrm{operator}\right)={\left(V\left(S.L.O\right)/n\right)}^{1/2}$$

(12) $$s_{T}\left(\mathrm{within}-\mathrm{laboratory}\right)={\left[V\left(O.L\right)+\left(V\left(S.L.O\right)/n\right)\right]}^{1/2}$$

(13) $$s_{T}\left(\mathrm{between}-\mathrm{laboratory}\right)={\left[V\left(L\right)+V\left(O.L\right)+\left(V\left(S.L.O\right)/n\right)\right]}^{1/2}$$

where *n* is the number of observations by a single operator, V(S.L.O) is the variance for specimens with laboratories and operators, V(O.L) is the variance for operators within a laboratory, and V(L) is the variance for laboratories. The three variances V(L), V(O.L) and V(S.L.O) can be calculated in accordance with ASTM D 2906.

Table 7 shows the source data for maximum bending force from the intra-laboratory test.

For different numbers of observations by a single operator or operators within the laboratory, for each average (*n*), such as 1, 3, 5 and 7, the critical differences for the maximum bending force ($F_{BM}$) for the four kinds of samples are listed in Table 8.

Two averages of observations should be considered significantly different at the 95% probability level if the difference equals or exceeds the critical differences shown in Table 8.

Using similar analysis methods and calculating procedures, the critical differences of single-operator and within-laboratory precision for maximum heat flux value, static friction index, and compression resilience index of thermal transfer, friction, and compression properties can be calculated and determined, as shown in Table 9, Table 10 and Table 11.

## 6. Conclusions

A new test method and a prototype mechanical measurement system called the Material Tactile Tester (MTT) were proposed and developed to measure and evaluate the tactile properties of porous polymeric sheet materials such as textile materials in consideration of dynamic interactions as complex as the contact process and all aspects of tactile sensation. The measurement system can automatically measure the thermal transfer, bending, friction and compression performances of porous polymeric materials during the dynamic contact between the measuring heads and the materials in one instrument, and is capable of associating the objective measurements with the subjective tactile sensations.

Derived from the test data and measurement curves, a series of indices were defined and graded to objectively characterize the tactile properties. A neural network prediction model for translating objective test results to subjective sensations of tactile properties was constructed based on objective and subjective test results.

Seven types of porous polymeric sheet materials from seven categories for the tactile properties were cut to a size of 200 mm × 200 mm and tested using MTT and KES system. Each index of tactile properties was significantly different (*P* < 0.05) between different sheet materials. The analysis of the correlations with the Kawabata KES test method showed that the correlations were significant when comparing maximum heat flux value, bending rigidity, intensity index of friction, and compression work of pressure release stage of the MTT tests with the Kawabata KES tests of thermal conductivity, hysteresis of bending moment, mean friction coefficient, and linearity of compression.

An intra-laboratory test was conducted for precision analysis, and four kinds of samples with different structural feature and made from different materials were measured. Critical differences for the maximum bending force, maximum heat flux, static friction index and compression resilience index of tactile properties were calculated, and single-operator precision and within-laboratory precision were determined based on the intra-laboratory test results.

The new test method and the mechanical measurement system provide objective characterization of tactile properties of porous polymeric materials for new product development and industrial applications.

## Figures and Tables

**Table 1:** Indices definition for objective evaluation of tactile properties.

Tactile	Symbol	Description	Unit
Thermal transfer	$H_{M}$	Maximum heat flux value	kW/m^2^
	$PSI_{Des}$	Psychosensory intensity of descending stage	
	$PSI_{Asc}$	Psychosensory intensity of ascending stage	
Bending	$F_{BM}$	Maximum bending force	mN
	$W_{BD}$	Bending rigidity	N·s
	$W_{BA}$	Bending recovery rigidity	N·s
Friction	SFI	Static friction index	
	DFI	Dynamic friction index	
	FII	Intensity index of friction	N·s
Compression	$W_{CD}$	Compression work of pressure stage	mN·cm/cm^2^
	$W_{CA}$	Compression work of pressure release stage	mN·cm/cm^2^
	CRI	Compression resilience index	

**Table 2:** Structural parameters of seven typical samples for objective and correlation tests.

Sample	Weight (g/m^2^)	Thickness (mm) at 4.14 kPa	Content
A	119.425	0.39	Polyester
B	191.05	4.93	Polyethylene foam
C	323.775	0.81	Linen
D	139.95	0.45	Polyester
E	75.4	3.05	Polyurethane foam
F	128.95	0.36	95% polyester + 5% spandex
G	2703.5	2.98	Silicone rubber

**Table 3:** Objective indices of tactile properties for correlation analysis between MTT system and KES system.

Tactile Properties	MTT System Indices	KES System Indices
Dynamic heat transfer characteristics	Maximum heat flux value $H_{M}$	Thermal conductivity K
Bending properties	Bending rigidity $W_{BD}$	Bending moment B
Friction properties	Intensity index of friction FII	Mean friction coefficient MIU
Compression properties	Compression work of pressure release stage $W_{CA}$	Linearity of compression LC

**Table 4:** Basic structural features of test samples for the precision test.

Sample	Weight (g/m^2^)	Thickness (mm) at 4.14 kPa
1	779.85	1.53
2	113.875	0.31
3	28.1	0.04
4	1089.875	13.64

**Table 5:** MTT test results of tactile properties by mean values.

Sample		*H_M_*	*W_B__D_*	*FII*	*W_C__A_*
A		0.24	5.64	33.6	67.3
B		0.31	14.26	73.3	39.2
C		0.40	6.7	21.4	75.5
D		0.093	8.64	26.6	77.1
E		0.33	9.32	70.2	34.5
F		0.32	3.87	27.2	27.6
G		0.16	15.17	23.6	29.5
One-way ANOVA	P (Sig.)	0.00	0.00	0.00	0.00

**Table 6:** KES test results of tactile properties by mean values.

Sample		K	B	MIU	LC
A		9.05	0.088	0.27	0.30
B		10.24	0.40	0.52	0.36
C		15.64	0.16	0.17	0.23
D		3.54	0.23	0.20	0.28
E		11.69	0.29	0.59	0.37
F		13.41	0.002	0.23	0.40
G		6.78	0.46	0.22	0.45
One-way ANOVA	P (Sig.)	0.00	0.00	0.00	0.00

**Table 7:** Raw data for the maximum bending force from the intra-laboratory experiment.

Sample	Operator	Test1	Test2	Test3	Test4	Test5
1	01	679.26	679.28	679.54	679.01	679.88
	02	679.01	678.93	678.99	679.12	678.93
2	01	35.83	34.37	35.67	35.56	35.02
	02	35.89	38.11	36.79	37.19	36.87
3	01	20.46	19.38	20.33	19.67	19.87
	02	18.76	22.07	19.76	20.88	19
4	01	1196.74	1197.81	1196.99	1197.5	1197.09
	02	1196.48	1197.51	1196.49	1197.08	1197.7

**Table 8:** Critical differences of maximum bending force for bending properties.

Number of Observations in Each Average	Sample 1		Sample 2		Sample 3		Sample 4	
	Single Operator	within Laboratory	Single Operator	within Laboratory	Single Operator	within Laboratory	Single Operator	within Laboratory
1	0.663	0.980	1.957	3.730	2.599	2.599	1.383	1.383
3	0.383	0.817	1.130	3.370	1.501	1.501	0.798	0.798
5	0.296	0.780	0.875	3.293	1.162	1.162	0.618	0.618
7	0.250	0.764	0.740	3.260	0.982	0.982	0.523	0.523

**Table 9:** Critical differences of maximum heat flux vale for thermal transfer properties.

Number of Observations in Each Average	Sample 1		Sample 2		Sample 3		Sample 4	
	Single Operator	within Laboratory	Single Operator	within Laboratory	Single Operator	within Laboratory	Single Operator	within Laboratory
1	1.842	6.524	0.613	4.230	1.228	5.228	1.162	1.162
3	1.063	6.348	0.354	4.201	0.709	5.131	0.671	0.671
5	0.824	6.313	0.274	4.195	0.549	5.111	0.520	0.520
7	0.696	6.297	0.232	4.192	0.464	5.103	0.439	0.439

**Table 10:** Critical differences of static friction index for friction properties.

Number of Observations in Each Average	Sample 1		Sample 2		Sample 3		Sample 4	
	Single Operator	Within Laboratory	Single Operator	Within Laboratory	Single Operator	Within Laboratory	Single Operator	Within Laboratory
1	0.00166	0.00264	0.00310	0.0149	0.00572	0.00872	0.00428	0.0116
3	0.00096	0.00227	0.00179	0.0147	0.00330	0.00736	0.00247	0.0110
5	0.00074	0.00218	0.00139	0.0146	0.00256	0.00706	0.00191	0.0109
7	0.00063	0.00215	0.00117	0.0146	0.00216	0.00693	0.00162	0.0109

**Table 11:** Critical differences of compression resilience index for compression properties.

Number of Observations in Each Average	Sample 1		Sample 2		Sample 3		Sample 4	
	Single Operator	within Laboratory	Single Operator	within Laboratory	Single Operator	within Laboratory	Single Operator	within Laboratory
1	0.0134	0.0200	0.0196	0.0196	0.00301	0.0123	0.0138	0.0577
3	0.00775	0.0167	0.0113	0.0113	0.00174	0.0120	0.00795	0.0566
5	0.00601	0.0160	0.00875	0.00875	0.00135	0.01195	0.00616	0.0564
7	0.00508	0.0157	0.00739	0.00739	0.00114	0.01193	0.00520	0.0563
